# A Lipidomic Approach to Identify Potential Biomarkers in Exosomes From Melanoma Cells With Different Metastatic Potential

**DOI:** 10.3389/fphys.2021.748895

**Published:** 2021-11-18

**Authors:** Simona Lobasso, Paola Tanzarella, Francesco Mannavola, Marco Tucci, Francesco Silvestris, Claudia Felici, Chiara Ingrosso, Angela Corcelli, Patrizia Lopalco

**Affiliations:** ^1^Department of Basic Medical Sciences, Neuroscience, and Sense Organs, University of Bari Aldo Moro, Bari, Italy; ^2^Department of Biomedical Sciences and Human Oncology, University of Bari Aldo Moro, Bari, Italy; ^3^Division of Medical Oncology, A.O.U. Consorziale Policlinico di Bari, Bari, Italy; ^4^Centre of Omic Sciences, IRCCS San Raffaele Hospital, Milan, Italy; ^5^Institute for Chemical and Physical Processes (IPCF)-CNR SS Bari, Bari, Italy

**Keywords:** melanoma, membrane vesicles, osteotropism, lipids, MALDI-TOF/MS

## Abstract

Melanoma, one of the most lethal cutaneous cancers, is characterized by its ability to metastasize to other distant sites, such as the bone. Melanoma cells revealed a variable *in vitro* propensity to be attracted toward bone fragments, and melanoma-derived exosomes play a role in regulating the osteotropism of these cells. We have here investigated the lipid profiles of melanoma cell lines (LCP and SK-Mel28) characterized by different metastatic propensities to colonize the bone. We have purified exosomes from cell supernatants by ultracentrifugation, and their lipid composition has been compared to identify potential lipid biomarkers for different migration and invasiveness of melanoma cells. Matrix-assisted laser desorption ionization-time-of-flight/mass spectrometry (MALDI-TOF/MS) lipid analysis has been performed on very small amounts of intact parental cells and exosomes by skipping lipid extraction and separation steps. Statistical analysis has been applied to MALDI mass spectra in order to discover significant differences in lipid profiles. Our results clearly show more saturated and shorter fatty acid tails in poorly metastatic (LCP) cells compared with highly metastatic (SK-Mel28) cells, particularly for some species of phosphatidylinositol. Sphingomyelin, lysophosphatidylcholine, and phosphatidic acid were enriched in exosome membranes compared to parental cells. In addition, we have clearly detected a peculiar phospholipid bis(monoacylglycero)phosphate as a specific lipid marker of exosomes. MALDI-TOF/MS lipid profiles of exosomes derived from the poorly and highly metastatic cells were not significantly different.

## Introduction

Living cells release lipid bilayer vesicles into the extracellular fluid, i.e., extracellular vesicles (EVs), which aroused great interest in the scientific community in the last 10 years due to their important role in complex intercellular communication.

Extracellular vesicles are released by cells of most living organisms (animals, bacteria, and plants) and are different in origin, size, and composition. They are grouped in microvesicles, apoptotic bodies, and exosomes. Microvesicles ranged between 150 and 1,000 nm in diameter and are formed by outward budding of the plasma membrane, while apoptotic bodies (over 1,000 nm) are released when plasma membrane blebbing occurs during apoptosis. Exosomes refer to smaller vesicles ranging from 30 to 150 nm and differ because of their endocytic origin. Endocytosis from the plasma membrane leads to the formation of an early endosome in the cytosol, and then the inward budding of the endosomal membrane fills its lumen with intraluminal vesicles, which mature in late endosome, termed also as multivesicular body (MVB). Their fusion with the plasma membrane allows for the release of intraluminal vesicles into the extracellular space while their different origin determines variation in lipid bilayer membrane. Their common feature, however, is that they carry a pool of functional biomolecules (including DNA, RNAs, proteins, and lipids) (Record et al., 2014; Isola et al., 2016; Kalluri, 2016; Kalra et al., 2016; Skotland et al., 2017; Kalluri and LeBleu, 2020).

Exosomes were described for the first time in 1983 in a study about the externalization of the transferrin receptor during maturation of sheep reticulocytes (Pan and Johnstone, 1983). It has been well-established that they are only produced from viable cells and play an important role in cell-to-cell communication through the transfer of their cargo.

Although both normal and pathological cells are able to secrete exosomes, cancer cells generally secrete more exosomes than normal counterparts (Tickner et al., 2014). Exosomes derived from cancer cells modify local and distant microenvironments and promote metastasis differently, as influencing the immune system, promoting epithelial to mesenchymal transition (EMT), angiogenesis, and organotropism (Zhang and Yu, 2019). The evidence of an exosomal role in cancer is widely reported, but the complex process by which exosomes promote oncogenic progression and metastases is yet to be clarified. Cancer-derived exosomes may establish a favorable milieu for cancer progression by carrying oncogenic cargo of proteins (Rajagopal and Harikumar, 2018). Some comparative studies on protein content between metastatic and non-metastatic exosomes have been reported thus revealing different protein expressions (Peinado et al., 2012; Garnier et al., 2013; Jeppesen et al., 2014).

It is noteworthy that high synthesis activity of the lipids is required for the proliferation of tumor cells to generate new biological membranes. Although the physiological mechanisms are not yet understood, the lipid synthesis in malignant tissues plays a critical role during tumorigenesis as the cell transformation, development, and tumor progression (Baenke et al., 2013).

On the other hand, the lipid characterization of different tumor cell lines has demonstrated the enrichment of some lipid species in contrast to the decrease of others, as compared to normal cells (Hilvo et al., 2011; Lee et al., 2012; Bandu et al., 2018; Messias et al., 2018; Szlasa et al., 2020). In this context, an important role is attributed to the higher level of saturated and monounsaturated phospholipids in cancer cells that can prevent lipid peroxidation and thus protect them from oxidative damage (Rysman et al., 2010).

It remains to be clarified how changes in lipid composition can affect the metastatic potential of cancer cells. In order to shed light on this, a detailed analysis of tumor tissue is required to determine the full spectrum of lipids within tumor cells. To this end, lipidomic approaches are often applied to analyze the lipid composition of tumor cell lines or fresh tumor samples by mass spectrometry (Hilvo et al., 2011; Bandu et al., 2018). Nevertheless, lipidomic characterization of tumor-derived exosomes, including melanoma, is still poorly investigated.

Melanoma cells, characterized by their ability to metastasize to distant sites, create a favorable environment for their growth by activating the EMT and favoring the immune system evasion (Sceneay et al., 2013; Tucci et al., 2014; Passarelli et al., 2017).

In a recent study, Mannavola et al., have investigated the potential role of melanoma-derived exosomes in favoring the motility and invasiveness of cells toward bone fragments by activating the SDF-1/CXCR4/CXR7 chemotactic axis under the influence of SDF-1 gradient. The authors found that poorly (LCP) and highly (SK-Mel 28) metastatic melanoma cells had variable propensity to be attracted toward the bone *in vitro*. As the migratory capacity of LCP cells increased once exposed to the stimuli of bone fragments, they are defined as “osteotropic,” by contrast, as bone fragments exerted only a modest effect on SK-Mel28 cells, they are named “not-osteotropic.” Hence, tumor-derived exosomes reprogram the “innate” osteotropism of melanoma cells by upregulating the membrane receptor CXCR7 (Mannavola et al., 2019).

This study aims at exploring the lipid content of both LCP and SK-Mel28 melanoma cells and exosomes in order to identify potential lipid biomarkers for their different migration behavior and invasiveness. We compare the lipid content of whole melanoma cells and exosomes by direct matrix-assisted laser desorption ionization-time-of-flight/mass spectrometry (MALDI-TOF/MS) lipid analysis of intact membranes. This approach is crucial for investigating by statistics analysis exosome membrane lipids. In parallel, the classical semiquantitative analysis of extracted lipids by TLC coupled to MS has been completed with the purpose to improve knowledge derived from a direct method and, especially, to clearly identify the peculiar phospholipid pattern in melanoma-derived exosomes.

## Materials and Methods

### Materials

All organic solvents used for lipid extraction and MS analyses were commercially distilled and of the highest available purity (Sigma–Aldrich). The matrix used for MALDI-TOF/MS analyses was the 9-Aminoacridine hemihydrate (9-AA) and was purchased from Acros Organics (Morris Plains, NJ, United States). The following commercial glycerophospholipids (used as standards): 1,2-dioleoyl-*sn*-glycero-3-phospho-(1′-rac-glycerol), bis(monooleoylglycero)phosphate, 1-palmitoyl-2- linoleoyl-*sn*-glycero-3-phosphate, bis(monomyristoylglycero) phosphate, N-palmitoyl-D-erythro-sphingosylphosphorylcho- line, N-stearoyl-D-erythro-sphingosylphosphorylcholine, *sn*-(3- myristoyl-2-hydroxy)-glycerol-1-phospho-*sn*-3′-(1′,2′-dimyrist- oyl)-glycerol, 1,2-dimyristoyl-sn-glycero-3-phospho-(1′-rac-gly- cerol), 1,2-dimyristoyl-*sn*-glycero-3-phosphate, 1,2-dimyristoyl- *sn*-glycero-3-phospho-L-serine, 1,2-diphytanoyl-*sn*-glycero-3-phosphoethanolamine, 1′,3′-bis[1,2-dimyristoyl-*sn*-glycero-3-phospho]-*sn*-glycerol, 1′,3′-bis[1,2-dioleoyl-*sn*-glycero-3-phos- pho]-*sn*-glycerol, were purchased from Avanti Polar Lipids, Inch (Alabaster, AL, United States). Plates for thin-layer chromatography (TLC) (Silica gel 60A, 10 × 20 cm, 0.2-mm-thick layer), were purchased from Merck (Darmstadt, Germany).

### Exosomes Isolation From Melanoma Cells and Characterization

Melanoma (SK-Mel28 and LCP) cell lines (ATCC, Rockville, MD, United States) were cultured in an exosome-free complete medium as previously described (Mannavola et al., 2019).

Exosomes were purified by ultracentrifugation of supernatants from 48-h cultured melanoma cells at 80% confluency (Théry et al., 2006). Extracellular vesicles were isolated from a 100-ml culture medium using, initially, three different sedimentation speeds: 300 × *g* for 10 min to remove cells, 2,000 × *g* to remove dead cells and debrides, and 10,000 × *g* to remove microvesicles. Next, the supernatant was centrifuged at 100,000 × *g* for 70 min two times at 4°C to collect exosomes. They were then resuspended in PBS and stored at −80°C.

After isolation, the characterization of exosomes has been performed exactly following the methods as previously described in Mannavola et al. (2019). Briefly, exosome preparations were verified by measuring the expression of CD63, CD81 (eBioscience), and CD9 (BD Pharmingen) by flow-cytometry (Tucci et al., 2018; Pezzicoli et al., 2020). Moreover, to further validate the purity of exosome preparations, western blots were performed to measure the levels of CD81, TSG101, calnexin, and bovine serum albumin in accordance with Minimal Information for Studies of Extracellular Vesicles (MISEV) guidelines (Théry et al., 2018). The transmission electron microscopy (TEM) defined the morphology of vesicles.

### Lipid Extraction

Total lipids of LCP and SK-Mel28 cells and exosomes were extracted by the Bligh and Dyer method (Bligh and Dyer, 1959); the extracts were carefully dried under N_2_ before weighing and then dissolved in chloroform (10 mg/ml). Usually, about 1 mg of total lipids was obtained from about 13 million cells of each cell line. In the present study, we have analyzed lipids extracted from three different preparations for each tumor cell line.

### Thin-Layer Chromatography Analyses (TLC)

Total lipid extracts were analyzed by TLC on silica gel 60A plates (Merck, 20 × 10 cm, layer thickness, 0.2 mm). The plates were washed two times with chloroform/methanol (1:1, by vol.) and activated at 180°C before use. Polar lipids were eluted with an acid solvent (chloroform/methanol/acetic acid/water, 85:15:10:3.5, by vol.). Total lipid detection was carried out by molybdenum blue spray reagent (Sigma-Aldrich) specific for phospholipids (Kates, 1986). Alternatively, total lipid detection was done with reversible staining exposing the TLC plate to iodine vapor for 4–5 min for staining all classes of lipids before the lipid bands isolation. To analyze in detail the various components of the lipid extracts, each band present on the plates was scraped and lipids extracted from silica, as previously described (Kates, 1986); briefly, 0.5 ml of a mixture chloroform/methanol/water (1:2:0.8, by vol.) has been added to silica bands, and the samples were vigorously stirred and centrifuged. Lipid bands of preparative TLC were analyzed by positive and negative ion mode MALDI-TOF/MS.

### Matrix-Assisted Laser Desorption Ionization-Time-of-Flight/Mass Spectrometry (MALDI-TOF/MS)

MALDI-TOF mass spectra were generally acquired in the negative and positive ion modes on a Bruker Microflex LRF mass spectrometer (Bruker Daltonics, Bremen, Germany). The system utilized a pulsed nitrogen laser, emitting at 337 nm, the extraction voltage was 20 kV, and gated matrix suppression was applied to prevent detector saturation. For each mass spectrum, 2,000 single laser shots (sum of 4 × 500) were averaged. The laser fluence was kept about 5% above threshold to have a good signal-to-noise ratio. All spectra were acquired in a reflector mode (detection range: 200–2,000 mass/charge, *m/z*) using the delayed pulsed extraction; spectra were acquired in negative and positive ion modes. Peaks areas, spectral mass resolutions, and signal-to-noise ratios were determined by the software for the instrument *Flex Analysis 3.3* (Bruker Daltonics).

A mix containing 1,2-dimyristoyl-sn-glycero-3-phosphate, 1,2-distearoyl-sn-glycero-3-phospho-(1′-rac-glycerol), sn-(3- myristoyl-2-hydroxy)-glycerol-1-phospho-sn-3′-(1′,2′-dimyris- toyl)-glycerol, 1′,3′-bis[1,2-dimyristoyl-sn-glycero-3-phospho]-sn-glycerol, GM1 Ganglioside was always spotted next to the sample as external standard, and an external calibration was performed before each measurement in a negative ion mode; the mass range of the authentic standards is 590–1,550 atomic mass units (amu). A mix containing 1,2-distearoyl-sn-glycero-3-phosphocholine, 1,2-dimyristoleoyl- sn-glycero-3-phosphocholine, 1,2-di-O-phytanyl-sn-glycero-3-phosphocholine was always spotted next to the sample as external standard, and an external calibration was performed before each measurement in a positive ion mode; the mass range of the authentic standards is 670–1,350 atomic mass units (amu).

For the analysis of lipid extract, the samples for MALDI-TOF analysis were prepared as previously described (Sun et al., 2008). Briefly, the total lipid extracts (10 mg/ml) were diluted from 20 to 200 μl with a 60:40 (by vol.) 2-propanol/acetonitrile mixture. Next, 10 μl of a diluted sample was mixed with 10 μl of 9-AA (10 mg/ml). Then, 0.35 μl of the mixture was spotted on the MALDI target.

Lipids from intact melanoma cells and exosomes produced were directly analyzed by the “intact method,” as previously described (Angelini et al., 2012). In brief, the cellular suspensions were syringed and centrifuged at 100,000 × *g* for 1 h and 10 min; the samples were all diluted to 0.5 μg/μl of total cellular protein concentration, determined by the Bradford method. Afterward, 1 μl of cellular suspension was spotted on the MALDI target (Micro Scout Plate, MSP 96 ground steel target). After water evaporation, a thin layer (0.35 μl) of matrix solution (9-AA 20 mg/ml in 2-propanol/acetonitrile, 60/40, by vol.) is then spotted in the dried sample. Finally, even after evaporation of the matrix, it is possible to analyze the sample directly with MALDI-TOF/MS.

In our study, mass spectra of three independent biological samples (i.e., three cell cultures and the corresponding lipid extracts) were considered to confirm reproducibility of the results.

In particular, series of MALDI mass spectra (three replicates for each sample) have been averaged by using the software for the instrument *CliProTools* 3.0 (Bruker Daltonics) in order to find the area under the peaks. The samples were analyzed, comparing different series of spectra from the two cell lines and the exosomes preparations. A *p*-value from paired Student’s *t*-test < 0.05 was set as the threshold to define significant differences between the series of spectra.

Post Source Decay (PSD) spectra were acquired on a Bruker Microflex mass spectrometer (Bruker Daltonics, Bremen, Germany), as previously described (Fuchs et al., 2007). Briefly, the precursor ions were isolated using a time ion selector. The fragment ions were refocused onto the detector by stepping the voltage applied to the reflectron in appropriate increments. This was done automatically by using the “FAST” (fragment analysis and structural TOF) subroutine of the Flex Analysis software. Mass accuracy of our instrument is 200 ppm (external calibration). A specific lipid database (Lipid Maps Database, LIPID MAPS Lipidomics Gateway) (LIPID MAPS) was used to facilitate and confirm the assignment of lipid species.

## Results

**FIGURE 1:**
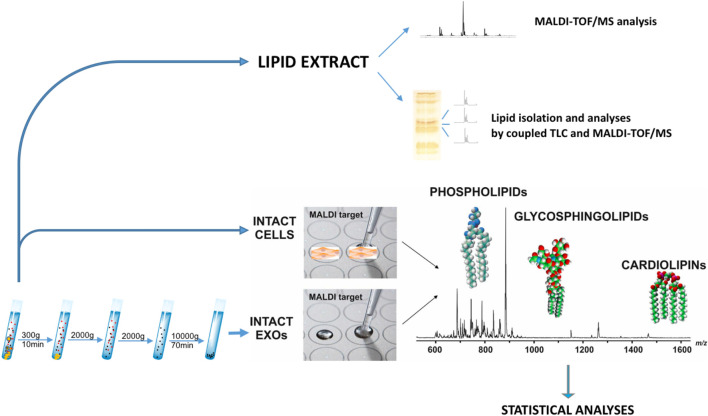
Lipidomics workflow for melanoma cells and exosomes. Lipid extract from melanoma cells has been analyzed by MALDI-TOF/MS and coupled TLC and MALDI-TOF/MS. After exosomes have been purified from supernatants of LCP and SK-Mel28 cells, small amounts of intact exosomes and cells were loaded on the MALDI target and lipids were directly analyzed by MS in order to perform statistical analysis.

The overall workflow employed here for the comprehensive lipidome analysis of LCP and SK-Mel28 cell lines and exosomes is shown in Figure 1. As previously described, total lipids of LCP and SK-Mel28 cells were extracted by the classical extraction method and analyzed by both MALDI-TOF/MS and coupled TLC and MALDI-TOF/MS.

In general, although main lipids are detectable by MALDI-TOF/MS, some lipid classes are more sensitively detected than others; consequentially, the signals of less sensitively detected phospholipids are suppressed, when complex mixtures are analyzed. Therefore, a pre-chromatographic separation of different lipid classes by TLC, followed by MALDI-TOF/MS analysis of individual lipid bands, offers the opportunity to identify minor components as well, whose signals were barely distinguishable from the noise in the total lipid profiles.

Then, in order to perform statistical analysis, MALDI-TOF/MS was used to directly acquire lipid profiles of the two melanoma cell lines and their purified exosomes. This “intact method” is highly sensitive and crucial to analyze only minute amounts of biological material; moreover, it is possible to directly load 1 μl only of an intact sample (at 0.5 μg/μl protein concentration) on the MALDI target, having a fast and detailed lipidomic analysis, reducing the processing time of the samples (Angelini et al., 2012).

### Analysis of Total Lipid Extracts of Melanoma Cell Lines by Coupled TLC and MALDI-TOF/MS

In order to detect main lipid species, those that ionize better in a negative ion mode (i.e., acidic lipid classes) and others that give intense signals in a positive ion mode (i.e., zwitterionic glycerophospholipids), depending on their chemical structures, we acquired the MALDI mass spectra of the total lipid extracts of melanoma cells in both negative and positive ion ionization modes. By comparing the MALDI lipid profiles of the two cell lines, we could not observe qualitative differences (Supplementary Figure 1).

**FIGURE 2:**
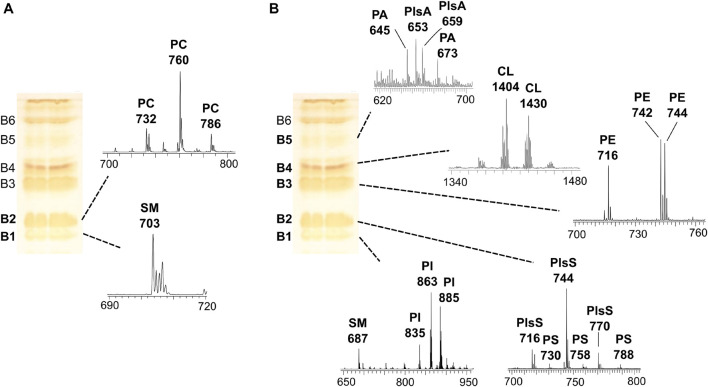
MALDI-TOF/MS analysis of individual lipid bands isolated from melanoma cells by TLC. The total lipid extract of cells (LCP cell line) was loaded on the plate (120 μg per each lane); TLC was stained with iodine vapors (temporary staining of all classes of lipids). Six lipid bands (B1–B6) were marked with a pencil, and silica was scraped; lipids were extracted from silica and analyzed by MALDI-TOF/MS. The same TLC plate is shown on the left of both panels. **(A)** shows the MALDI-TOF/MS spectra of the lipid bands acquired in positive ion mode, while **(B)** shows the mass spectra of the lipid bands acquired in negative ion mode.

In order to gain detailed information on lipid species, the coupled TLC and MALDI-TOF/MS analysis was performed. First, the total lipid extract was analyzed by TLC, and lipids were stained by iodine vapors in order to isolate and purify the individual components (see plates on the left of the Figures 2A,B). Then, the various lipid bands were isolated by preparative TLC and analyzed by MALDI-TOF/MS (mass spectra of lipid bands are on the right of the plates in Figures 2A,B). By TLC analyses, individual lipids were identified by comparison of their retention factor (R_f_) values with those of standards and by their response to specific staining (not shown).

In particular, six bands (B1–B6) of lipids were scraped from the plate, and then lipids were extracted from silica (Bligh and Dyer, 1959) and analyzed by MALDI-TOF/MS in positive and negative ion modes.

Figure 2A shows the MALDI-TOF mass spectra of lipid bands B1 and B2 in a positive ion mode, while Figure 2B shows mass spectra of bands B1–B5 in a negative ion mode.

A detailed list of main peaks detected in the MALDI-TOF mass spectra acquired in negative and positive ion modes, corresponding to the lipid species present in the lipid bands and/or in the total lipid extracts, is reported in Tables 1, 2, respectively.

**TABLE 1:** Assignments of *m/z* values detected in negative ion mode of lipids mode MALDI-TOF mass spectra of lines and exosomes.

*m/z* value	Assignment [M-H]^–^
478.3	LPE (18:1)
480.3	LPE (18:0)
506.3	LPE (20:1)
645.4	PA (32:1)
646.4	PlsE (P-30:0)/PlsE (O-30:1)
653.3	PlsA (P-34:3)/PlsA (O-34:4)
659.5	PlsA (P-34:0)/PlsA (O-34:1)
673.4	PA (34:1)
687.6	SM (16:0)
697.3	PA (36:3)
701.5	PA (36:1)
714.5	PE (34:2)
716.5	PE (34:1)/PlsS (P-32:1)
718.5	PE (34:0)
722.4	PE (P-36:4)
730.5	PS (32:2)
742.5	PE (36:2)
744.6	PE (36:1)/PlsS (P-34:1)/PlsS (O-34:2)
750.5	PlsE (P-38:4)
758.4	PS (34:2)
760.4	PS (34:1)
766.5	PE (38:4)
768.5	PE (38:3)
769.6	BMP (36:4)
770.5	PE (38:2)/PlsS (P-36:2)/PlsS (O-36:3)
771.7	BMP (36:3)
772.6	PE (38:1)
786.6	PS (36:2)
788.6	PS (36:1)
797.7	BMP (38:4)
799.7	BMP (38:3)
833.5	PI (34:2)
835.6	PI (34:1)
861.5	PI (36:2)
863.6	PI (36:1)
883.5	PI (38:5)
885.5	PI (38:4)
887.6	PI (38:3)
911.5	PI (40:5)
913.6	PI (40:4)
1151.8	GM3 (16:0)
1179.8	GM3 (18:0)
1235.9	GM3 (22:0)
1261.9	GM3 (24:1)
1263.9	GM3 (24:0)
1399.9	CL (68:4)
1404.0	CL (68:2)
1425.9	CL (70:5)
1430.0	CL (70:3)

*The numbers (x:y) denote the total length (as carbon numbers) and number of double bonds of acyl chains, respectively.*

**TABLE 2:** Assignments of *m/z* values detected in positive ion mode MALDI-TOF mass spectra of melanoma cell lines and exosomes.

*m/z* value	Assignment [M+H]^+^
494.4	LPC (16:1)
496.3	LPC (16:0)
522.3	LPC (18:1)
550.5	LPC (20:1)
703.5	SM (16:0)
706.5	PC (30:0)
720.6	PlsC (O-32:0)
725.5	SM (16:0) (+ Na^+^)
732.5	PC (32:1)
734.5	PC (32:0)
742.5	PlsC (P-34:2)/PlsC (O-34:3)
746.6	PlsC (P-34:0)/PlsC (O-34:1)
758.5	PC (34:2)
760.5	PC (34:1)
786.5	PC (36:2)
788.6	PC (36:1)
810.5	PC (38:4)

*The numbers (x:y) denote the total length (as carbon numbers) and number of double bonds of acyl chains, respectively.*

The sphingomyelin (SM) was detected in the band of the lowest retention factor on TLC (B1): positive ion mode MALDI-TOF/MS analysis of B1 revealed a main peak at *m*/*z* 703.5, while negative ion mode analysis of the same band revealed a signal at *m*/*z* 687.6 (see B1 mass spectra in Figures 2A,B). Both the signals correspond to the same species of SM, having a palmitic acid.

B1, analyzed in a negative ion mode, shows peaks attributable to the phospholipid phosphatidylinositol (PI), where the main peaks are at *m*/*z* 835.6, 863.6, and 885.5 referable to different PI species (Figure 2B).

The mass spectrum of B2, in a positive ion mode, shows main peaks at *m*/*z* 732.5, 760.5, and 786.5, corresponding to protonated form of phosphatidylcholine (PC) species with different chain lengths (Figure 2A). A MALDI negative ion mode of the same band B2 shows peaks attributable to phosphatidylserine (PS) and some plasmenylserine (PlsS) species; the peaks at *m*/*z* 716.5, 744.6, and 770.5 can be assigned to the PlsS species, while the peaks at *m*/*z* 730.5, 758.4, and 788.6 refer to PSs (Figure 2B).

B3, analyzed in a negative ion mode, shows peaks (at *m*/*z* 716.5, 742.5, and 744.6) attributable to phosphatidylethanolamine (PE) species (Figure 2B).

In the mass spectrum of B4, in a negative ion mode, the peaks in the high range *m/z* 1,300–1,500 were assigned to cardiolipin (CL) species, having four acyl chains (at *m/z* 1404 and 1430) (Figure 2B).

B5, analyzed in a negative ion mode, shows low signals (at *m*/*z* 645.4 and 673.4) attributable to phosphatidic acid (PA) species; the peaks at *m*/*z* 653.3 and 659.5 can be assigned to plasmalogens species (PlsA).

B6 was assigned to the neutral lipid cholesterol (CHO) by comparing its R_f_ value on TLC with that of lipid standard, although we could not confirm its identity by MALDI-TOF/MS.

The use of coupled TLC and MALDI-TOF/MS analysis gave also the opportunity to identify and validate the assignments of minor lipids present in melanoma cells, such as PS, CL, and plasmalogen species. In conclusion, the TLC lipid profile of melanoma cells consists (in R_f_ order) of SM/PI, PS/PC, PE, CL, PA, and CHO.

### Comparative Lipid Analysis of Melanoma Cells and Exosomes by MALDI-TOF/MS

LCP and SK-Mel28 melanoma cell lines and exosomes have been analyzed by MALDI-TOF/MS using the intact method in order to perform statistical analysis of their lipid components.

Exosomes were purified from supernatants of SK-Mel28 and LCP cells at a final concentration of 0.2–1.1 × 10^11^ vesicles/ml. Supplementary Figure 2 illustrates specific characteristics of exosomes, including the histogram that represents the size distribution of nanovesicles purified from LCP-conditioned supernatants (Panel A), TEM images showed the typical cup-shaped morphology of exosomes (Panel B), the presence of CD81, CD63, or CD9 tetraspanins (Panel C), and, finally, the western blot analyses (Panel D) confirm that exosome preparations were positive for typical markers of EVs (CD81 and TSG101), while excluded the possible contamination by large-EVs (>200 nm) or non-EV structures, such as protein aggregates, since all the samples resulted negative for both CANX and BSA as compared to the complete control medium.

#### MALDI Negative Ion Mode Analysis

**FIGURE 3:**
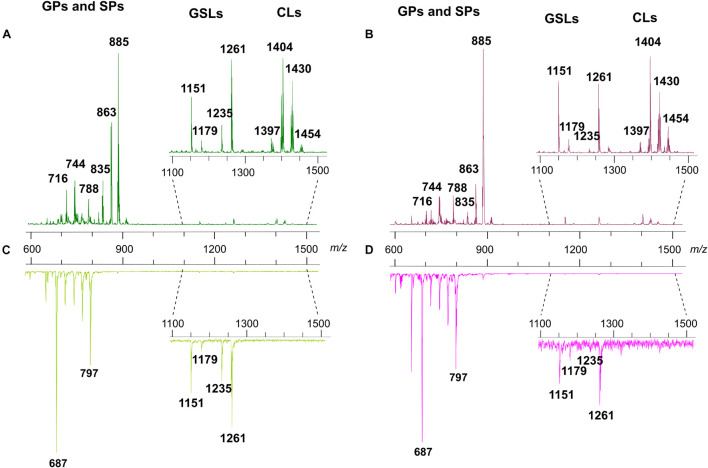
Negative ion mode MALDI-TOF/MS lipid profiles of two melanoma cell lines and the derived exosomes. The upper spectra show the typical lipid profiles of LCP **(A)** and SK-Mel28 **(B)** cells by the intact method. The lower spectra show the typical lipid profiles of intact exosomes derived from LCP **(C)** and SK-Mel28 **(D)** cells. The enlargements of range *m/z* 1,100–1,500 of mass spectra are also shown. GPs, glycerophospholipids; SPs, sphingolipids; GSLs, glycosphingolipids; CLs, cardiolipins.

Figure 3 shows the comparison between the representative lipid profiles of two melanoma cell lines, LCP and SK-Mel28 in Panels A and B, respectively, and those of exosomes they produce in Panels C and D, respectively, obtained by negative ion mode MALDI-TOF/MS analysis.

By comparing the four panels, it can be seen that, although not perfectly overlapping, the lipid profiles of the two cell samples (Figures 3A,B) share many similarities as well as no qualitative differences can be observed between the lipid patterns of the LCP-derived exosomes and SK-Mel28-derived exosomes (Figures 3C,D, respectively).

The main signals in the MALDI-TOF mass spectra, attributable to the negative [M-H]^–^ molecular ions of all lipid classes here detected, are collected in Table 1.

The main peaks in both mass spectra of melanoma cells (Figures 3A,B) are visible in the range of major glycerophospholipids. The signals at *m*/*z* 835.6, 863.6, and 885.5 are assigned to various PI species. We also performed PSD analysis of the higher signal 885, which highlights the presence of an arachidonic acid (C20:4) and a stearic acid (C18:0) in the molecular structure of PI 38.4 (not shown). The peaks at *m*/*z* 716.5 and 744.6 could be attributable both to PE and PlsS species, while the peak at *m*/*z* 788.6 corresponds to PS. Other signals, attributable to various species of PI, PE, PA, SM, PS, and lysophosphatidylethanolamine (LPE) species, are also visible in both mass spectra as minor peaks (Table 1).

Additional peaks at *m*/*z* 1151.8, 1179.8, 1235.9, and 1261.9 can be observed in the *m*/*z* 1,100–1,500 enlargement of the mass spectra (Figures 3A,B, inset). These MALDI signals are compatible with complex acidic glycosphingolipids, carrying three sugar residues in their molecules and different fatty acids, named GM3. Furthermore, the minor peaks detected in the two lipid profiles of cells at *m*/*z* 1404.0 and 1430.0 correspond to the main CLs species (Figures 3A,B, inset).

As said before, the exosome lipid profiles in the negative ion mode of MALDI-TOF/MS are shown in the Figures 3C,D; both the lipid patterns are dominated by signals in the *m*/*z* range 600–800, compatible with major glycerophospholipid and sphingophospholipid species.

In the range of phospholipids, the major MALDI peaks are at *m/z* 687.6 and 797.7; the first signal is attributable to the sphingolipid SM, while the second one is attributable to the glycerophospholipid bis(monoacylglycero)phosphate (BMP).

The BMP molecules are similar to phosphatidylglycerol (PG) molecules in terms of mass, but the difference is in the bonding position of the two acyl chains: the two fatty acid chains are at *sn*-1 and *sn*-2 positions on the same glycerol backbone, in the PG structure; while, in the BMP structure, the two fatty acid chains are esterified at the sn-1 position of each glycerol (Holbrook et al., 1992; Hankin et al., 2015). This structural variation makes identification of PG and BMP lipid species *via* accurate mass alone almost impossible since they have the same exact molecular formula (Anderson et al., 2017).

Therefore, in order to elucidate the chemical identity of the high peak at *m*/*z* 797.7 in the mass spectra of exosomes, we have performed additional analyses. Supplementary Figure 3 shows the TLC lipid profile of exosomes where individual phospholipids were identified by comparison of their R_f_ value with those of lipid standards; it consists mainly of (in R_f_ order) lysophosphatidylcholine (LPC), SM, PS, PC, PE, PA, and BMP, with PC as the most abundant phospholipid in the membrane of exosomes. CL, the phospholipid marker of mitochondria, was not found by TLC analysis, in agreement with data obtained by MALDI-TOF analysis of exosomes, where no signals attributable to CL species were visible (see Figures 3C,D, inset).

As regards BMP, it is noteworthy that the higher (in R_f_ order) lipid spot of exosomes, having the same R_f_ value of BMP 36:2 standard, was assigned to BMP; whereas, the R_f_ value of PG 36:2 standard is different due to the different positions of the acyl chains linked to the glycerol backbone (Supplementary Figure 3).

Furthermore, PSD analysis of the peak at *m*/*z* 797.7 (shown in Supplementary Figure 4) validates the chemical structure of BMP 38:4 constituted of stearic acid (C18:0) and arachidonic acid (C20:4) as fatty acid tails.

Finally, both the MALDI mass spectra of exosomes shown in Figures 3C,D present some signals corresponding to other glycerophospholipids species, such as PA, PE, PS, and PI, as minor peaks. Furthermore, smaller signals assigned to GM3 species are visible in the *m/z* 1,100–1,300 enlargement (see Figures 3C,D, inset).

**FIGURE 4:**
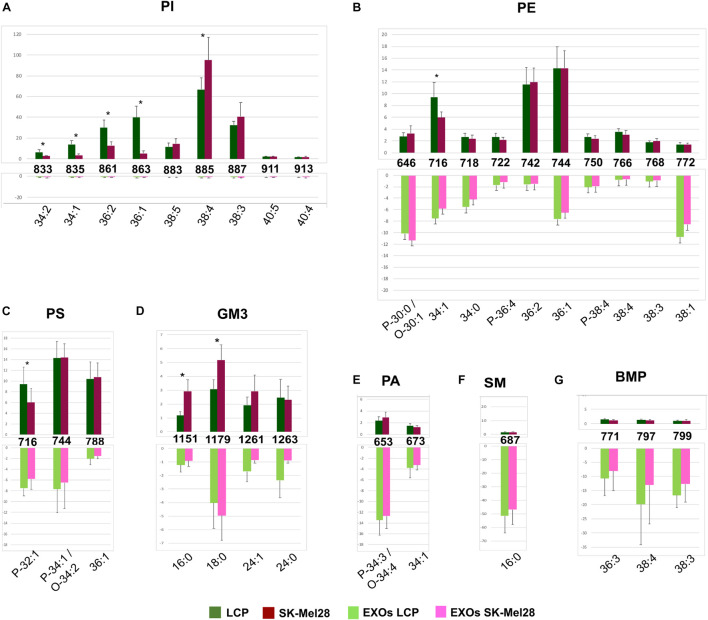
Significant differences of (–) MALDI signals in lipid profiles of two cell lines (upper panels) and the derived exosomes (lower panels). The histograms **(A–G)** show the significant differences in intensity between the lipid peaks present in the four series of (–) mass spectra. *p*-value < 0.05 was set as the threshold to define significant differences. Significant differences between the signals detected in the cell lines profiles (shown in the upper panels) are also highlighted by an asterisk. Data are reported as the average value of intensity ± SD. The numbers reported between upper and lower panels indicate the MALDI *m/z* signals. Lipid assignments for each signal are also indicated.

Significant differences in intensity of MALDI signals detected in the negative ion mass spectra of melanoma cells (upper panels) and exosomes (lower panels) are reported as histograms in Figures 4A–G. First, by comparing series of replicates of LCP and SK-Mel28 cell lines mass spectra, we found that the following peaks at *m*/*z* 833.5, 835.6, 861.5, and 863.6, attributable to PI species 34:2, 34:1, 36:2, and 36:1, respectively, were significantly higher in the LCP sample (Figure 4A upper panel). Whereas the peak at *m*/*z* 885.5 (corresponding to PI 38:4) was significantly higher in SK-Mel28 than the LCP sample (Figure 4A, upper panel), suggesting that PI species with shorter acyl chains and having one and two double bonds are more abundant in the LCP than in the SK-Mel28 cell line.

No other significative differences in MALDI negative ion mode analyses between the two cell lines were observed, except an higher peak at *m*/*z* 716.5, corresponding to PE 34:1 (Figure 4B, upper panel) and PlsS P-32:1 in the LCP sample (Figure 4C, upper panel), and two higher peaks, at *m*/*z* 1151.8 and 1179.8, assigned to GM3 16:0 and 18:0, respectively, in the SK-Mel28 sample (Figure 4D, upper panel).

The MALDI peaks corresponding to the main PI, PE, and PS species (Figures 4A–C) present in exosomes mass spectra were significantly lower than those present in their parental cells. It is worth noting that only the signals corresponding to saturated and monounsaturated PE species (P-30:0/O-30:1, 34:0, and 38:1) were significantly higher in the exosomes than in their parental cells (Figure 4B). As regards GM3 species, the signals (at *m*/*z* 1151.8, 1261.9, and 1263.9) assigned to GM3 16:0, 24:1, and 24:0, respectively, were significantly lower in the MALDI lipid profile of SK-Mel28-derived exosomes compared to those present in parental cells (Figure 4D); while no significant difference in GM3 signal intensities has been observed between the lipid profiles of LCP-derived exosomes and their parental cells (Figure 4D).

Furthermore, the MALDI peaks corresponding to PA P-34:3/O-34:4 and 34:1 (at *m*/*z* 653.3 and 673.4, respectively) (Figure 4E), SM 16:0 (at *m*/*z* 687.7) (Figure 4F) and BMP 36:3, 38:4, and 38:3 (at *m*/*z* 771.7, 797.7, and 799.7) (Figure 4G) were significantly higher in the lipid profiles of exosomes compared with those present in their parental cells mass spectra.

In conclusion, by comparing the two lipid profiles of melanoma cells with variable metastatic propensity, we observed more saturated and shorter fatty acid tails in poorly metastatic (LCP) cells compared with highly metastatic (SK-Mel28) cells, particularly for some species of PI, whereas the content of polyunsaturated PI 38:4 and GM3 species with saturated acyl chains increases in the SK-Mel28 cells.

Finally, no significant differences in the MALDI signals were observed between the lipid profiles of the two exosome preparations.

#### MALDI Positive Ion Mode Analysis

**FIGURE 5:**
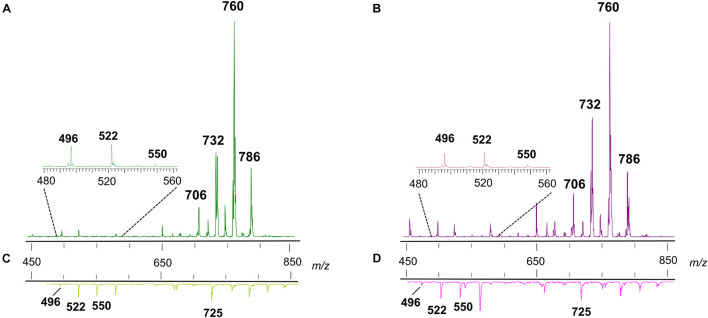
Positive ion mode MALDI-TOF/MS lipid profiles of two melanoma cell lines and the derived exosomes. The upper spectra show the typical lipid profiles of LCP **(A)** and SK-Mel28 **(B)** cells by the intact method. The lower spectra show the typical lipid profiles of intact exosomes derived from LCP **(C)** and SK-Mel28 **(D)** cells. The enlargements of range *m/z* 480–560 of mass spectra are also shown.

Figure 5 shows the comparison between the representative lipid profiles of the two melanoma cell lines LCP and SK-Mel28, in Panels A and B, respectively, and those of exosomes they produce in Panels C and D, respectively, obtained by positive ion MALDI-TOF/MS analyses; it can be seen that the higher peaks are present in the *m*/*z* range 600–800 of the lipid profiles, while smaller peaks are detectable in the lower *m/z* range 400–600. The main signals in the MALDI-TOF mass spectra, attributable to the positive [M+H]^+^ molecular ions of glycerophospholipids and sphingophospholipids, are collected in Table 2.

In both the melanoma cells mass spectra (Figures 5A,B), the peaks at *m*/*z* 706.5, 732.5, 734.5, 760.5, and 786.5 attributable to the molecular ions [M+H]^+^ of PC species are predominant. Furthermore, various plasmenylcholine (PlsC) species were barely detected as minor peaks. Smaller MALDI signals at *m*/*z* 496.3, 522.3 and 550.5 are visible in the enlargements of both the mass spectra, and are compatible with the molecular ion [M+H]^+^ of LPC species (Figures 5A,B, inset).

In the lipid mass spectra of the two exosome preparations, shown in Figures 5C,D, the MALDI signals compatible with PC species are not dominant, while their intensities are similar to those of LPC species.

Furthermore, the molecular species corresponding to SM 16:0 was found in both the exosome lipid profiles as a signal at *m*/*z* 725.5, corresponding to the sodiated form of the molecule, also previously described by negative ion mode MALDI analysis (peak at *m/z* 687 in Figures 3C,D).

**FIGURE 6:**
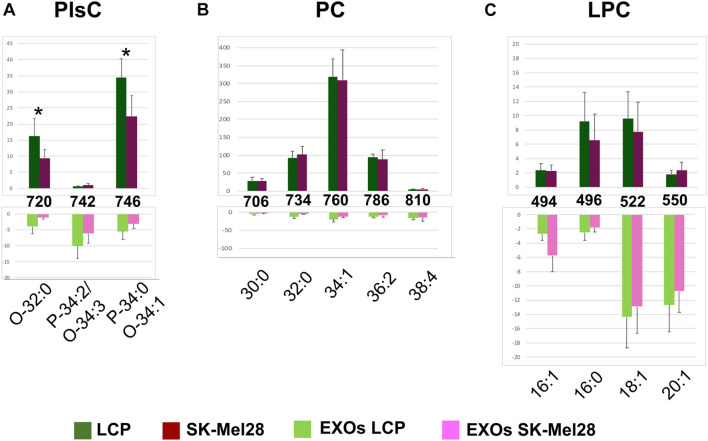
Significant differences of (+) MALDI signals in lipid profiles of two cell lines (upper panels) and the derived exosomes (lower panels). The histograms **(A–C)** show the significant differences in intensity between the lipid peaks present in the four series of (+) mass spectra. *p*-value < 0.05 was set as the threshold to define significant differences. Significant differences between the signals detected in the cell lines profiles (shown in the upper panels) are also highlighted by an asterisk. Data are reported as the average value of intensity ± SD. The numbers reported between upper and lower panels indicate the MALDI *m/z* signals. Lipid assignments for each signal are also indicated.

Figures 6A–C show significant differences in intensity of MALDI peaks detected in the positive ion mass spectra of melanoma cells (upper panels) and exosomes (lower panels).

First, by comparing series of replicates of LCP and SK-Mel28 cell lines mass spectra, we found that the following peaks were significantly higher in the LCP sample: PlsC O-32:0 and PlsC P-34:0/O-34:1 (at *m*/*z* 720.6 and 746.6, respectively) (Figure 6A, upper panel). No other significant differences in peak intensities were found between the lipid profiles of the two cell lines (see Figures 6B,C, upper panels).

As regards differences between exosomes and parental cells, the signal at *m*/*z* 742.5, assigned to the molecular ion [M+H]^+^ of PlsC P-34:2/O-34:3, was significantly higher in the exosomes (Figure 6A); while those corresponding to PlsC O-32:0 and P-34:0/O-34:1 were significantly lower (see histograms in Figure 6A).

The signals at *m*/*z* 706.5, 734.5, 760.5, and 786.5, assigned to the molecular ions [M+H]^+^ of PC 30:0, 32:0, 34:1, and 36:2 species, respectively, were significantly higher in the lipid profiles of melanoma cells, being very lower in those of exosomes. Only the signal corresponding to PC 38:4 (at *m*/*z* 810.5) was significantly higher in the exosomes than in the parental cells (see histograms in Figure 6B).

Regarding the LPC species, only the peak at *m*/*z* 496.3, corresponding to saturated species LPC 16:0, was significantly lower in the exosomes than in their parental cells, while the peaks at *m*/*z* 494.4, 522.3, and 550.5, assigned to the monounsaturated species LPC 16:1, 18:1, and 20:1, respectively, were significantly higher in exosomes (see histograms in Figure 6C).

In conclusion, poorly metastatic cells contain higher levels of PlsC than highly metastatic ones; as regards to both exosomes, a decrease of PC and an increase of LPC content were observed in comparison to parental cells.

## Discussion

The present article is the first lipidomic-based study that characterizes exosomes derived from melanoma cell lines, having different metastatic potential, as previously characterized (Mannavola et al., 2019, 2020).

Although the MALDI-TOF/MS technique is not suitable for analyzing the fatty acids content in a biological sample, our results show that, in general, melanoma cells contain low levels of polyunsaturated phospholipid species, having fatty acid chains mainly constituted of 16 and 18 carbon atoms. Furthermore, the detected sphingolipid species, such as SM and GM3, were mainly constituted of saturated fatty acids (palmitic and stearic acids).

It is well-known that different chain lengths and degrees of unsaturation are able to affect the physical properties of cell membrane, either modifying its fluidity or stabilizing membrane proteins in the lipid domains (Szlasa et al., 2020). The question whether cell membrane rigidity could affect proliferation or metastasis of cancer cells has not yet been answered. Recently, it has been reported that the membrane fluidity and plasticity increased in cancer cells to facilitate their penetration into the blood vessels (Szlasa et al., 2020), and that a fluid membrane accelerates cell adhesion and metastatic capacity, for example, in breast cancer (Ross et al., 2016).

The differences of the composition in fatty acids between melanoma cells, having different metastatic potentials, and, in particular, the reduction of the degree of unsaturation and the length of the acyl chains in poorly metastatic cells here found, agree with previous reports describing melanoma cells with a reduced tumor cell migration, containing saturated phospholipids (Ross et al., 2016).

As regards PI, a progressive increase in some species with saturated and monounsaturated fatty acyl chains was associated with melanoma metastasis and progression, and these lipid species can be considered novel biomarkers for estimating the metastatic ability of melanoma cells (Kim et al., 2017). Although our findings on the higher level of PI 38:4 (containing both arachidonic and stearic acid) in highly metastatic melanoma cells disagree with the previous evidence, it can be also considered that a higher content of polyunsaturated fatty acids increases the membrane fluidity of these cells.

The higher content of C20:4 in highly metastatic melanoma cells could correlate with progression and metastatic behavior cells. In general, phospholipid species containing C20:4 can be hydrolyzed by phospholipase A2, and the produced C20:4 is converted into prostaglandin E2 (PGE2) by cyclooxygenase-2 and PGE synthase (Wang and DuBois, 2010). It is noteworthy that the levels of these species containing C20:4 were decreased in melanoma cells compared with those in normal cells and were associated with increase in PGE2, which contributes to the development, progression, and metastasis of cells (Kim et al., 2017).

As regards the content of the main glycosphingolipid detected, the enrichment in GM3 containing saturated fatty acids in the highly metastatic melanoma cells is in contrast with its general suppressive effect on cancer development and progression (Hakomori and Handa, 2015). GM3 is highly enriched in a type of membrane microdomain termed “glycosynapse” and forms complexes with co-localized cell-signaling molecules, including certain proteins tetraspanins enriched in exosomes membranes. GM3 modulates cell adhesion, growth, and motility by altering molecular organization in glycosynaptic microdomains and the activation levels of co-localized-signaling molecules that are involved in cancer pathogenesis (Hakomori and Handa, 2015). Moreover, it has been reported that GM3 downregulates the invasiveness capacity of human bladder cancer cells, while it can prevent haptotactic cell migration in colorectal cancer cell lines (Palacios-Ferrer et al., 2021). Further studies will be necessary to elucidate possible roles of this lipid in metastasis progression of melanoma.

Another interesting aspect concerns plasmalogens, which represent up to 20% of the total phospholipid mass in humans and appear to be associated with common disorders and diseases, including cancer (Braverman and Moser, 2012; Messias et al., 2018). In general, cancer cells are enriched in alkyl and alk-1-enyl ether lipids compared with normal cells, enough to be considered as a potential diagnostic marker for some species of cancer (Fernandes et al., 2020). Thanks to our MALDI-TOF lipid analyses, we detected various Pls species (such as PlsE, PlsS, PlsA, and PlsC) in both melanoma cell lines; noteworthy is the significant decreased content of PlsC O-32:0 and PlsC P-34:0/O-34:1 species in the highly metastatic cells. We can speculate that, in particular, the lower content of PlsC could be related to high oxidative stress associated with cancer progression, according to previous evidence on ovarian cancer cells, where decreased PlsC levels have been reported and correlated with oxidative stress (Hou et al., 2016).

As it was previously reported that LCP-derived exosomes were able to induce osteotropism in SK-Mel28 cells (Mannavola et al., 2019), we have also investigated if the quantitative differences of the lipid species observed between the two cell lines matched with any differences in the exosomes they produce.

It is well-known that exosomes carry bioactive lipids, which trigger cell-to-cell signaling, but the lipid-related aspects of exosomes have not obtained sufficient attention in the scientific literature.

Some studies have shown an enrichment of lipid species, including CHOL, SM, glycosphingolipids, and PS in exosome membranes; in contrast, exosomes generally contained lower levels of PC and PI than their parental cells, and only small changes were reported for PE content (Baenke et al., 2013; Record et al., 2014; Lydic et al., 2016; Skotland et al., 2017, 2019). This change in lipid composition apparently increases the exosomes ability to fuse with neighboring cells (Tickner et al., 2014). Lipidomics data appear to be inconsistent, and there is poor knowledge about lipid profiling of exosomes from melanoma cells.

These data are the first lipidomic analysis of exosomes derived from LCP and SK-Mel28 melanoma cells by MALDI-TOF/MS in positive and negative ion modes. No significant differences between the two lipid profiles of exosomes are observed in our experiments, but, as expected, the intensities of some signals of lipids in the mass spectra of exosomes are very different from those of their parental cells.

In agreement with previous reports, our statistical analyses of the MALDI lipid profiles of both exosome preparations clearly show an enrichment in the sphingolipid SM, but also in other glycerophospholipid species, such as PA, LPC, and BMP, compared with their parental cells. It is worth noting that the levels of polyunsaturated species of PC and PlsC were significantly higher in the exosomes; as previously indicated, the high content of double bonds in the membrane lipids makes the exosome membrane more fluid, which is a fundamental characteristic in the fusion process of vesicles with neighboring cells. Besides, only few signals corresponding to PE species were significantly higher in the exosomes than in their parental cells. Furthermore, we show a clear decrease of the content of PI species in the two exosomes preparations compared to their parental cells. Also, other MALDI-TOF/MS signals corresponding to phospholipids PE, PS, PC, and PlsC species were significantly lower in exosomes.

A higher content in SM, PA, and LPC in exosome membranes compared to parental cells highlights the presence of membrane microdomains necessary for cell-cell communication and cell-signaling functions. The high level of LPC in exosomes could depend on its intracellular origin (i.e., endolysosomal compartment), but it can be also related to the role of this lysocompound as substrate for autotaxin, which is a lysophospholipase D that converts LPC in LPA (Moolenaar and Perrakis, 2011). It is known that autotaxin, to which exosomes can bind, is involved in the motility stimulation and has been also found in melanoma cells (Jethwa et al., 2016). It has been also suggested that the extracellular hydrolysis of phospholipids like LPC by metastatic tumor cells and the subsequent cellular uptake of the resulting free fatty acids seem to be a necessary prerequisite for metastatic potential of epithelial tumor cells, probably for generating pro-metastatic lipid second messengers (Raynor et al., 2015).

Moreover, it is interesting the intriguing presence of the polyglycerophospholipid BMP in exosomal membranes, since its presence in the vesicles is still controversial in the litterature. Its unusual lipid structure consists of two monoacylglycerols linked through one phosphate group; it is found in most mammalian cells and tissues, where it represents only 1% of cellular phospholipids, but it was found to be mostly enriched in lysosomes and endosomes. However, its presence in the exosomes is still unclear. This derives from the complexity of its identification that mostly depends by its modest content as well as the difficult discrimination from PG. Some authors have hypothesized that the amount and the distribution of BMP are cell type dependent, and/or BMP-enriched exosomes can be released under endolysosomal stress (Jabs et al., 2008; Miranda et al., 2018; Rabia et al., 2020).

To our knowledge, there are poor data on the presence of BMP in tumor cell-derived exosomes and, in particular, in those from melanoma. The combined lipid analysis by TLC and MALDI-TOF/MS allowed us to confirm the presence of various BMP species in melanoma exosomes, and statistical analysis leads us to conclude that it is significantly enriched compared with their parental cells. It can be added that the BMP enrichment in exosomes may be related to the biogenesis process rather than to its possible role in cancer progression.

Although we observed significant differences in some lipid species potentially implicated in the variable osteotropic propensity between the analyzed melanoma cells, we did not find any quantitative difference between the lipid components of the two exosome preparations.

To this regard, the limited sample size may have affected our experimental results. Further investigation, including *in vivo* isolation of exosomes, as well as parental tumor cells from either metastatic or not-metastatic melanoma patients, should be addressed to improve our lipid analysis.

On the other hand, we cannot exclude a biological reason for not identifying potential lipid biomarkers for different osteotropic behaviors of melanoma-derived exosomes. Has metastatic potential no influence on lipid sorting into exosomes? Do Exosomal lipids not mediate metastatic potential? Additional studies and analyses will be necessary to answer these crucial questions.

In conclusion, here, we used the direct MALDI-TOF/MS analysis, an extremely sensitive analytical technique, to achieve the most comprehensive lipid analysis of melanoma exosomes reported to date. Therefore, our data make a useful contribution to a comprehensive understanding of melanoma-secreted exosomes lipid molecular compositions. Understanding the precise physiological function of exosomes will be critical to determining their important role in cancer.

The basic knowledge of lipid species (quality and relative abundance) of the cell membranes (and in specific intracellular compartments) of melanoma cells might help in developing new pharmacological approaches to affect cell survival and reduce bone metastasis.

## Data Availability Statement

The raw data supporting the conclusions of this article will be made available by the authors, without undue reservation.

## Author Contributions

AC and FS designed the research. FM and MT provided samples. CF, PL, CI, FM, and PT performed the research. AC, SL, MT, and PL analyzed the data and wrote the manuscript. All authors contributed to the article and approved the submitted version.

## Conflict of Interest

The authors declare that the research was conducted in the absence of any commercial or financial relationships that could be construed as a potential conflict of interest.

## Publisher’s Note

All claims expressed in this article are solely those of the authors and do not necessarily represent those of their affiliated organizations, or those of the publisher, the editors and the reviewers. Any product that may be evaluated in this article, or claim that may be made by its manufacturer, is not guaranteed or endorsed by the publisher.

## Abbreviations

9-AA: 9-Aminoacridine hemihydrate
BMP: bis(monoacylglycero)phosphate
CL: cardiolipin
CHO: cholesterol
EMT: mesenchymal transition
EV: extracellular vesicles
GSLs: glycosphingolipids
GPs: glycerophospholipids
GSLs: glycosphingolipids
LPC: lysophosphatidylcholine
LPE: lysophosphatidylethanolamine
MALDI-TOF/MS: matrix-assisted laser desorption ionization-time-of-flight mass spectrometry
PA: phosphatidic acid
PC: phosphatidylcholine
PE: phosphatidylethanolamine
PG: phosphatidylglycerol
PI: phosphatidylinositol
Pls: plasmalogens
PS: phosphatidylserine
PSD: post source decay
R_f_: retention factors
SM: sphingomyelin
SPs: sphingophospholipids
TEM: transmission electron microscopy
TLC: thin-layer chromatography.
