# Supplementary material for: A Lipidomic Approach to Identify Potential Biomarkers in Exosomes From Melanoma Cells With Different Metastatic Potential
Source: Front Physiol. 2021 Nov 18;12:748895. doi: 10.3389/fphys.2021.748895 (PMC8637280; doi:10.3389/fphys.2021.748895)
Supplement: Supplementary file 1 [file Data_Sheet_1.ZIP › Supplementary Material/Fig.S4.pdf]

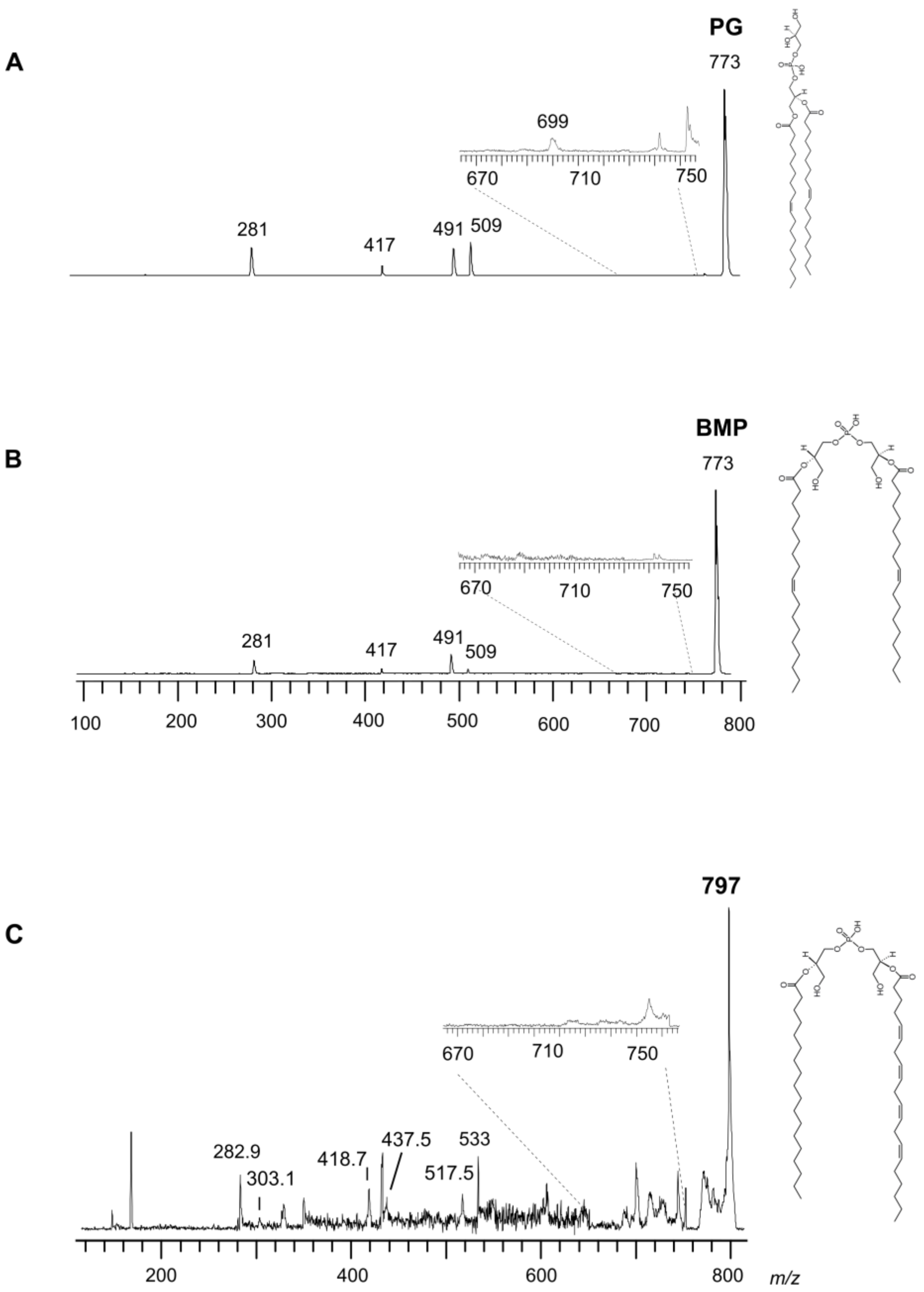

Fig. S4: Fragmentation patterns of lipid standards (PG 36:2 and BMP 36:2) (panel A and B, respectively) and molecular ion at *m/z* 797 (panel C) from SK-derived EXOs mass spectrum. Both mass spectra, corresponding to the phospholipid standards PG and BMP, have molecular ion $[M-H]^-$ at *m/z* 773.7 and a daughter ion at *m/z* 281.3, corresponding to oleic acid (18:1) as expected (panel A and B). In the *m/z* region 400-600 of both standard mass spectra, daughter ions at *m/z* 417.3, 491.3 and 509.5, corresponding to LPA-H_2O, LPG-H_2O and LPG, were observed (A and B). A daughter ion at *m/z* 699.5, corresponding to PA, produced by loss of glycerol from precursor ion, was observed for PG standard only, but not for BMP standard (enlargement in A and B).

In the PSD mass spectrum of molecular ion 797.5 of the sample (C) a daughter ion of mass 282.9, 303.1, 418.8 and 437.5 corresponding to stearic acid, arachidonic acid, LPA -H_2O and LPA, respectively, were observed, but the peak at *m/z* 723 corresponding to the loss of glycerol from precursor ion is absent (enlargement in C). This indicates that the structure of the lipid, corresponding to the peak at *m/z* 797.1 of our sample, is attributable to BMP (18:0/20:4), which does not have an esterified glycerol.
