# Supplementary material for: A Lipidomic Approach to Identify Potential Biomarkers in Exosomes From Melanoma Cells With Different Metastatic Potential
Source: Front Physiol. 2021 Nov 18;12:748895. doi: 10.3389/fphys.2021.748895 (PMC8637280; doi:10.3389/fphys.2021.748895)
Supplement: Supplementary file 1 [file Data_Sheet_1.ZIP › Supplementary Material/Fig.S3.pdf]

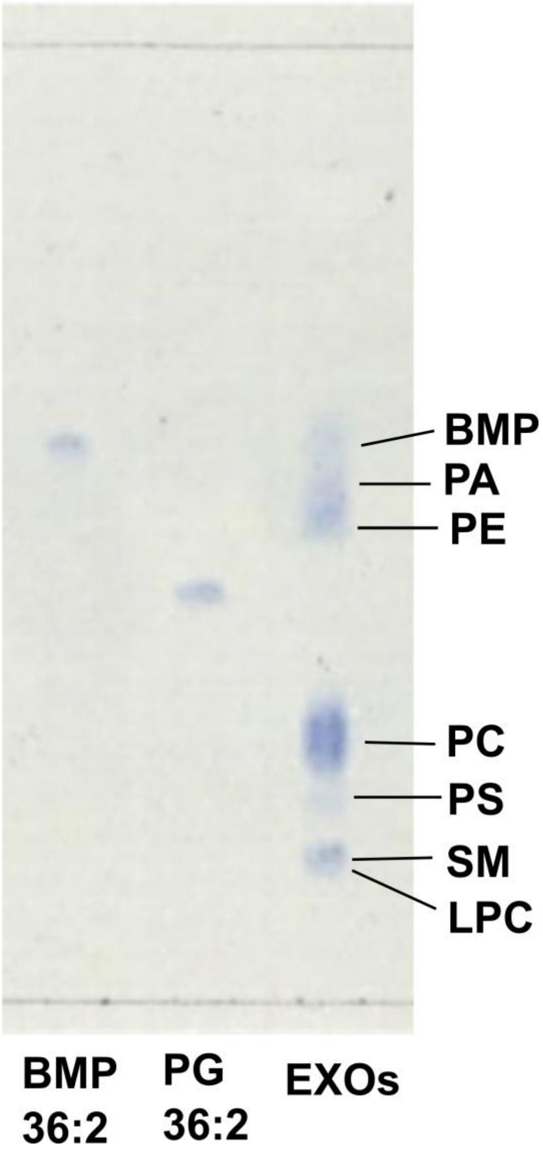

Fig. S3: Phospholipid TLC profile of EXOs. Total lipid extract of EXOs derived from LCP melanoma cells was eluted with acid solvent in order to separate polar lipids; then phospholipids were detected by spraying with molybdenum blue reagent. Sixty micrograms of lipid extract and five micrograms for each standard lipid were loaded on the TLC plate. BMP = bis(monoacylglycero)phosphate, PG = phosphatidylglycerol, PA = phosphatidic acid, PE = phosphatidylglycerol, PC = phosphatidylcholine, PS = phosphatidylserine, SM = sphingomyelin, LPC = lysophosphatidylcholine.
