# Supplementary material for: A Lipidomic Approach to Identify Potential Biomarkers in Exosomes From Melanoma Cells With Different Metastatic Potential
Source: Front Physiol. 2021 Nov 18;12:748895. doi: 10.3389/fphys.2021.748895 (PMC8637280; doi:10.3389/fphys.2021.748895)
Supplement: Supplementary file 1 [file Data_Sheet_1.ZIP › Supplementary Material/Fig.S2.pdf]

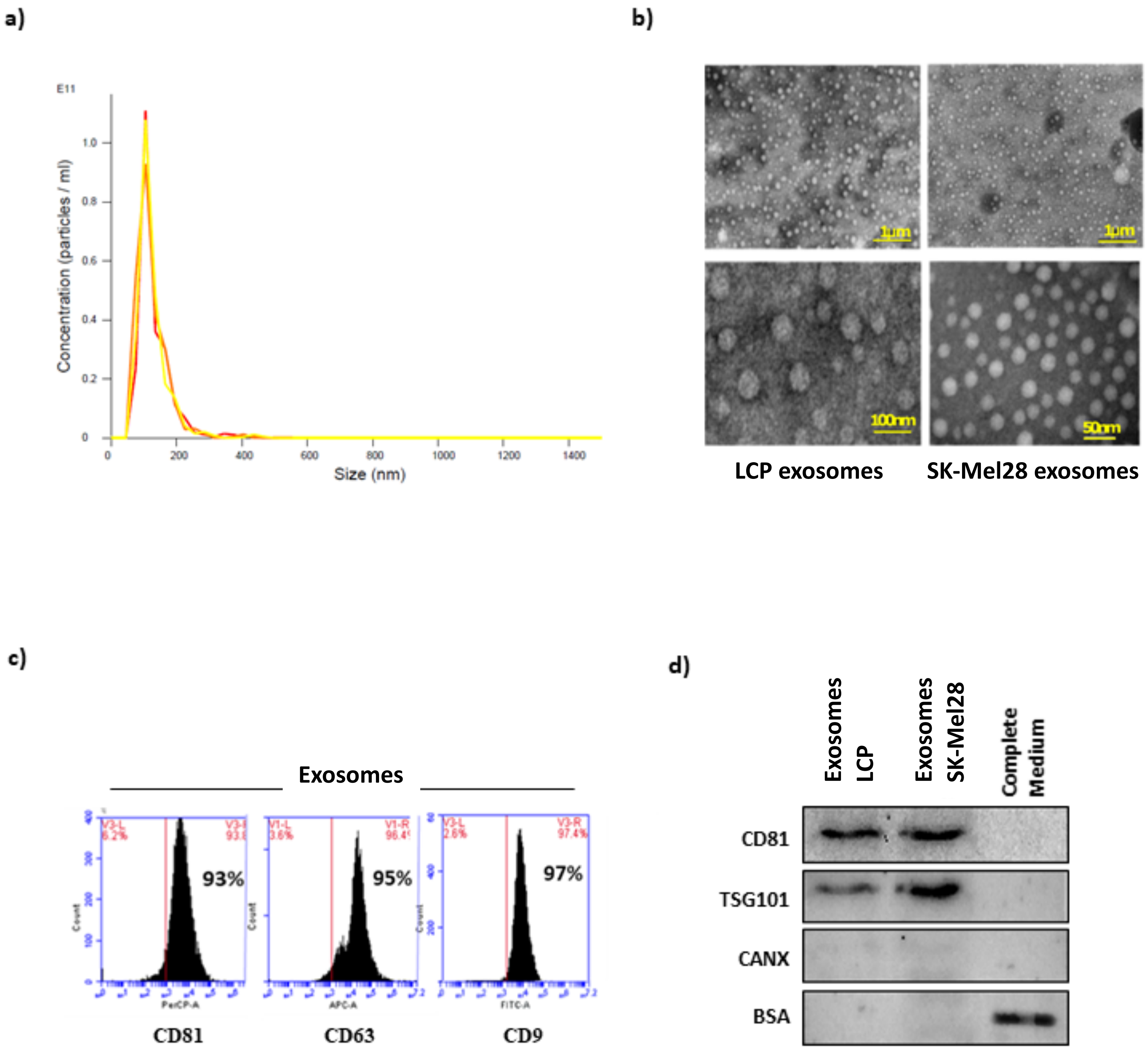

Figure S2: Characterization of exosomes from melanoma cells.
Exosome preparations were analyzed using NanoSight technology. Histogram represents the size distribution of nanovesicles purified from LCP conditioned supernatants, and the analysis reveals more than 80% of vesicles with a diameter ranging from 30 to 150 nm. Results are the mean from three different measurements (panel a). Representative panel showing exosome preparations by TEM reveals the presence of nanovesicles with typical cup-shaped morphology (panel b). Flow-cytometry shows the CD81, CD63 and CD9 tetraspanin expression in exosome preparations (panel c). Western blot for exosome typical markers (CD81 and TSG101) and potential contaminants (CANX, BSA). Fresh exosome-free complete medium was used as control (panel d).
