# Supplementary material for: A Lipidomic Approach to Identify Potential Biomarkers in Exosomes From Melanoma Cells With Different Metastatic Potential
Source: Front Physiol. 2021 Nov 18;12:748895. doi: 10.3389/fphys.2021.748895 (PMC8637280; doi:10.3389/fphys.2021.748895)
Supplement: Supplementary file 1 [file Data_Sheet_1.ZIP › Supplementary Material/Fig.S1.pdf]

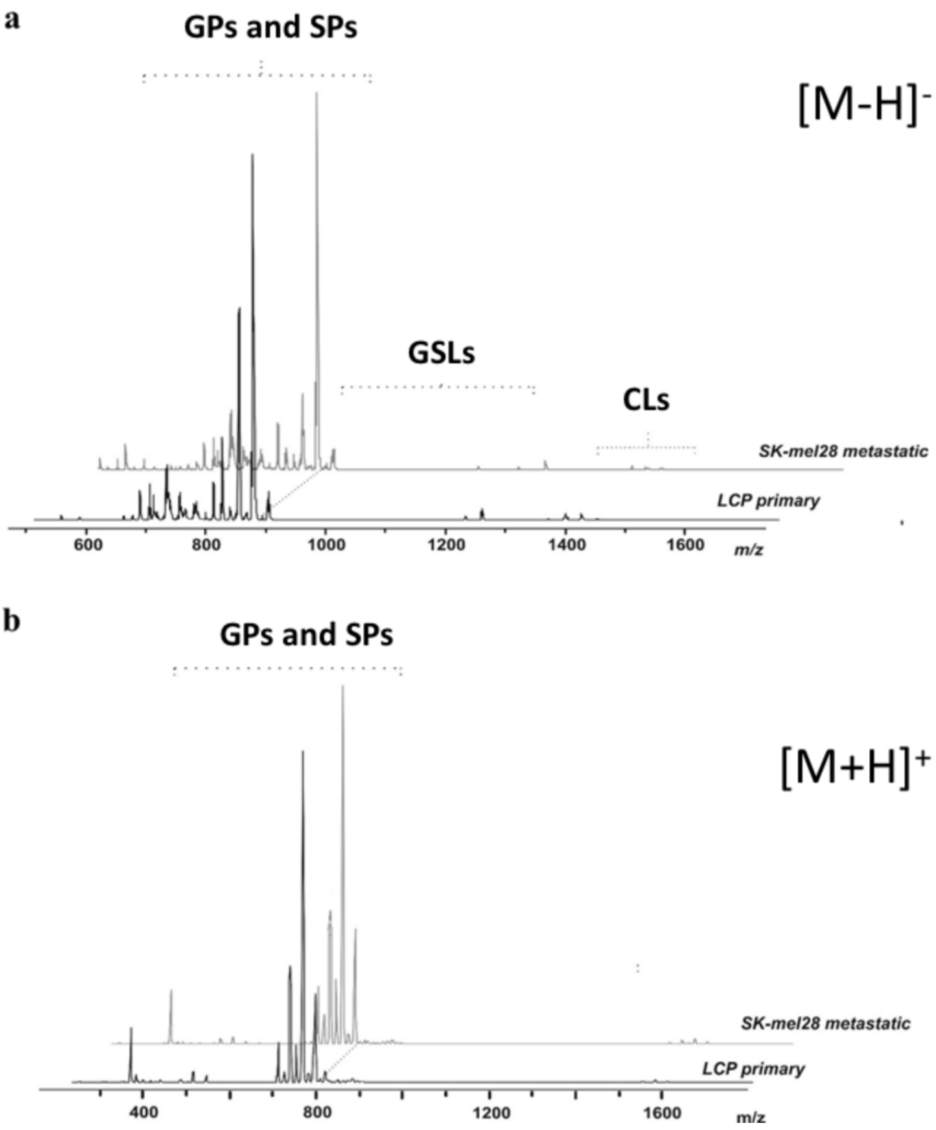

Fig. S1: (A) Negative and (B) positive ion mode MALDI-TOF/MS analyses of the lipid extracts of LCP and SK-Mel28 cells. In the figure the typical lipid profiles are shown. The signals present in the MALDI-TOF/MS lipid profiles, acquired in negative ion mode, can be grouped in two main *m/z* ranges: the *m/z* range 700–900 where the higher peaks attributable to glycerophospholipid (GPs) and sphingophospholipid (SPs) species are present, and the *m/z* range 1100–1500 where minor peaks compatible with glycosphingolipid (GSLs) and CLs species are visible. In the MALDI-TOF/MS lipid profiles, acquired in positive ion mode, the higher peaks are present in the *m/z* range 600–800 of the lipid profiles, while smaller peaks are detectable in the lower *m/z* range 400–600.
